# Ameliorative Effects of Grape Seed Proanthocyanidin Extract on Growth Performance, Immune Function, Antioxidant Capacity, Biochemical Constituents, Liver Histopathology and Aflatoxin Residues in Broilers Exposed to Aflatoxin B_1_

**DOI:** 10.3390/toxins9110371

**Published:** 2017-11-15

**Authors:** Shahid Ali Rajput, Lvhui Sun, Niya Zhang, Mahmoud Mohamed Khalil, Xin Gao, Zhao Ling, Luoyi Zhu, Farhan Anwar Khan, Jiacai Zhang, Desheng Qi

**Affiliations:** 1 Department of Animal Nutrition and Feed Science, College of Animal Science and Technology, Huazhong Agricultural University, Wuhan 430070, China; dr.shahidali@hotmail.com (S.A.R.); lvhuisun@mail.hzau.edu.cn (L.S.); zhangniya@mail.hzau.edu.cn (N.Z.); mahmoud.khalil@fagr.bu.edu.eg (M.M.K.); gaoxinnutrition@webmail.hzau.edu.cn (X.G.); lingzhao@webmail.hzau.edu.cn (Z.L.); zhuluoyi@outlook.com (L.Z.); hznyzjc@hotmail.com (J.Z.); 2 Animal Production Department, Faculty of Agriculture, Benha University, Moshtohor, Benha, Kalubia 13736, Egypt; 3 Department of Animal Health, Faculty of Animal Husbandry and Veterinary Sciences, University of Agriculture, Peshawar 25120, Pakistan; farhan82@aup.edu.pk

**Keywords:** Aflatoxin B_1_, grape seed proanthocyanidin extract, broilers, antioxidant capacity, detoxification, histopathology, residue

## Abstract

Aflatoxicosis is a grave threat to the poultry industry. Dietary supplementation with antioxidants showed a great potential in enhancing the immune system; hence, protecting animals against aflatoxin B_1_-induced toxicity. Grape seed proanthocyanidin extract (GSPE) one of the most well-known and powerful antioxidants. Therefore, the purpose of this research was to investigate the effectiveness of GSPE in the detoxification of AFB_1_ in broilers. A total of 300 one-day-old Cobb chicks were randomly allocated into five treatments of six replicates (10 birds per replicate), fed *ad libitum* for four weeks with the following dietary treatments: 1. Basal diet (control); 2. Basal diet + 1 mg/kg AFB_1_ contaminated corn (AFB_1_); 3. Basal diet + GSPE 250 mg/kg; (GSPE 250 mg/kg) 4. Basal diet + AFB_1_ (1 mg/kg) + GSPE 250 mg/kg; (AFB_1_ + GSPE 250 mg/kg) 5. Basal diet + AFB_1_ (1mg/kg) + GSPE 500 mg/kg, (AFB_1_ + GSPE 500 mg/kg). When compared with the control group, feeding broilers with AFB_1_ alone significantly reduced growth performance, serum immunoglobulin contents, negatively altered serum biochemical contents, and enzyme activities, and induced histopathological lesion in the liver. In addition, AFB_1_ significantly increased malondialdehyde content and decreased total superoxide dismutase, catalase, glutathione peroxide, glutathione-S transferase, glutathione reductase activities, and glutathione concentration within the liver and serum. The supplementation of GSPE (250 and 500 mg/kg) to AFB_1_ contaminated diet reduced AFB_1_ residue in the liver and significantly mitigated AFB_1_ negative effects. From these results, it can be concluded that dietary supplementation of GSPE has protective effects against aflatoxicosis caused by AFB_1_ in broiler chickens.

## 1. Introduction

Mycotoxins are the natural compounds that are produced by fungi, and aflatoxins are the most common known type of these mycotoxins, which are mainly produced by the *Aspergillus flavus* and *Aspergillus paraciticus* [1]. Aflatoxin B_1_ (AFB_1_) is considered to be the most highly widespread and toxic type of aflatoxins [2]. Feed contaminated with AFB_1_, either naturally or purified, can result in aflatoxicosis in poultry, and hence reduce growth performance and immunity, alter the blood biochemistry parameters, and intestinal morphology in broilers [3,4,5]. AFB_1_ increases the production of free radicals, augments the oxidative damage and lipid peroxidation, and hence leads to cell damage and death to animals or humans [6,7]. To further extend, AFB_1_ has been classified by the International Agency for Research on Cancer (IARC) as a group 1 carcinogens to humans, and it is known for its hepatotoxic, teratogenic, and immunosuppressive effects on humans and animals [8]. Moreover, the residues of AFB_1_ presented in animal products, such as meat, could result in serious health problems for humans. Also, there is a positive correlation between aflatoxin intake and human liver cancer, which has been demonstrated in various regions of Asia and Africa [9,10]. Oxidative stress has been reported to play a key role in the toxicity mechanism of AFB_1_; accordingly, the supplementing of antioxidants to animal feed have the ability to protect animals against AFB_1_-induced toxicity by enhancing the antioxidant system and immunity [11,12,13]. Proanthocyanidins are natural compounds that are found in plant based foods, (i.e., grape seed extract). Grape seed proanthocyanidin extract (GSPE) is an extract that is derived from grape seed, enriched with polyphenolic flavonoids, oligomeric proanthocyanidins, and polymerized oligomers. In China, GSPE has been widely used as a dietary supplement having tremendous health benefits to animals and humans [14,15]. For example, GSPE has a wide positive effects, as anti-mutagenic [16], cardioprotective [17,18], and neuroprotective [19] in previous experimental trails. Moreover, Liu et al. [20], reported that GSPE has the ability to enhance working memory, ameliorate symptoms of Alzheimer’s disease. Despite the fact that some antioxidants were reported to accelerate cancer progression in smokers and other people at high risk for lung cancer. GSPE is well-known as a powerful antioxidants in the world due to its ability to absorb oxygen radicals, as well as it has anti-inflammatory and anti-cancer effects [21,22]. GSPE has been reported to have significant protection effects against free radicals, free radical-induced lipid peroxidation, and DNA damage as compared to vitamins C, E, and A [23,24]. It has been reported that feeding Kunming mice on GSPE has a significant protective effect on zearalenone-induced hepatic injury and oxidative stress in liver [25]. Furthermore, GSPE increases the body weight gain, significantly improves the oxidative damage of the spleen, and alleviates the immune injury in mice induced by AFB_1_ [26]. It is well documented that GSPE can protect the functions of major organs by improving the antioxidant system, as well as prevent liver injury caused by carbon tetrachloride and ischemia/reperfusion [24,27]. Some researcher revealed that GSPE could prevent drug-induced liver and kidney damage, and can induce anti-tumor and anti-radiation activity [28,29]. Additionally, GSPE can alleviate arsenic-induced oxidative reproductive toxicity and protects the renal function from cisplatin-induced nephrotoxicity [30,31]. The protective action of GSPE against doxorubicin-induced adverse effects was further demonstrated by improving the antioxidants capacity [32]. Likewise, studies have shown that GSPE can be used as a potent antioxidant to improve the antioxidant system status and abnormalities of diabetic rats arising from streptozotocin [33].

Nevertheless, it is not clear whether supplementation of GSPE to AFB_1_ contaminated diets might detoxify aflatoxicosis by improving the oxidative status and the antioxidant defense system in broiler chickens. The objective of this study was to evaluate the toxic effects of AFB_1_ and the protective efficacy of GSPE on growth performance, serum biochemistry, serum immunoglobulins, liver histopathology, serum, and liver antioxidant enzymes activities and aflatoxin residues in the liver of broilers that are exposed to feed contaminated with aflatoxin B_1_.

## 2. Results

### 2.1. Growth Performance

The effects of dietary treatments on growth performance are summarized in Table 1. During the whole experimental period, the group fed with AFB_1_ contaminated diet (1 mg/kg) recorded the lowest average daily gain (ADG) and average daily feed intake (ADFI) (*p* < 0.05) as compared with other groups. This effect was alleviated by the addition of GSPE (250 and 500 mg/kg) into diets contaminated with AFB_1_, with a significant increase in ADG and ADFI when compared with the AFB_1_ group. Similarly, feed conversion ratio (FCR) of broilers was negatively affected by the dietary AFB_1_ group during the experimental period (*p* < 0.05). The supplementation of GSPE (250 and 500 mg/kg) resulted in markedly better FCR (*p* < 0.05) as compared with the AFB_1_ group. More importantly, the performance of broilers was not affected by the treatment with GSPE alone. These results demonstrated the effect of GSPE on eliminating the toxic effect of AFB_1_ on growth performance.

### 2.2. Serum Biochemistry

The current experiment revealed the effect of supplementing AFB_1_ contaminated diet with GSPE in different doses on serum biochemical changes. The results presented in Table 2 showed that feed contaminated with AFB_1_ (1 mg/kg), negatively affected (*p* < 0.05) the serum biochemical profile as compared with other groups. This toxic effect of AFB_1_ has been ameliorated by supplementation of GSPE (250 and 500 mg/kg), resulted in a significant decrease in the serum level of Alanine aminotransferase (ALT), Aspartate aminotransferase (AST), gamma-glutamyl transferase (GGT), and alkaline phosphatase (ALP) by nearly 31%, 16%, 16%, and 13%, respectively, when compared with the group fed AFB_1_ alone. Whereas, no significant differences were found when compared with the control group. On the same trend, the addition of 250 or 500 mg/kg GSPE to AFB_1_ contaminated diet, significantly improved the content of total protein (TP), albumin (ALB), and globulin (GLU) as compared with group fed with AFB_1_ contaminated diet alone. However, no significant differences were found in the biochemical parameters when GSPE 250 mg/kg supplemented to non-contaminated diet compared with the control group.

### 2.3. Serum Immunoglobulins

**Figure 1:**
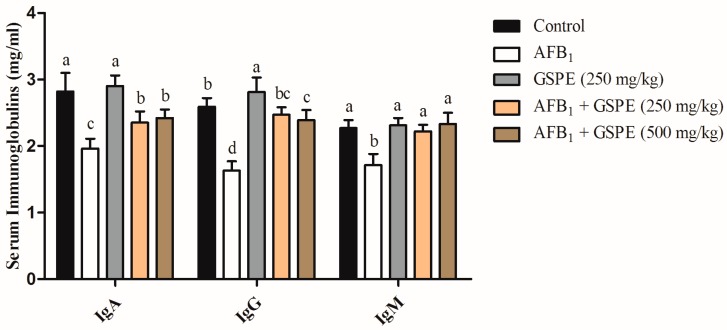
Effects of GSPE on serum immunoglobulins parameters of broilers fed diets contaminated with AFB_1_. Values are represented as the mean ± SD (*n* = 6). ^a–d^ column with different superscript letters were significantly different (*p* < 0.05). AFB_1_, aflatoxin B_1_; GSPE, grape seed proanthocyanidin extract.

From Figure 1, it has been cleared that the feeding broilers diet contaminated with AFB_1_ (1 mg/kg) altered the immune response of birds. AFB_1_ significantly (*p* < 0.05) reduced IgA, IgG, and IgM by 24%, 51%, and 36%, respectively, when compared with the control group. The addition of GSPE (250 or 500 mg/kg) to contaminated diet alleviated (*p* < 0.05) the toxic effect of AFB_1_ on serum immunoglobulin parameters. In contrast, there were no significant differences between the two levels of GSPE (250 and 500 mg/kg) when supplemented to contaminated diet. These results indicated that AFB_1_ caused damage to the immune system. However, the addition of GSPE to AFB_1_ contaminated diet was able to counteract the adverse effects of AFB_1_ on the immune system.

### 2.4. Serum Antioxidant Parameters

The effects of GSPE on the serum antioxidants indices of broiler exposed to AFB_1_ are summarized in Table 3. Broilers fed on a diet contaminated with AFB_1_ increased the serum malondialdehyde (MDA) content compared with the control group (*p* < 0.05). However, the addition of GSPE into diets contaminated with AFB_1_ significantly decreased the level of MDA content with no difference in the level of MDA between both doses (250 and 500 mg/kg) of GSPE groups. The activities of total superoxide dismutase (T-SOD), glutathione peroxide (GSH-Px), catalase (CAT), glutathione reductase (GR), glutathione-S transferase (GST), and the concentration of glutathione (GSH), were (*p* < 0.05) decreased with AFB_1_ treatment as compared with the control group. While the addition of GSPE (250 and 500 mg/kg) in AFB_1_ contaminated diet significantly improved the antioxidant enzymes activities. These results confirmed that GSPE significantly improved the antioxidant activities in the serum and decreased the oxidative damage induced by AFB_1_.

### 2.5. Histopathological Variations and Relative Weight of Liver

**Figure 2:**
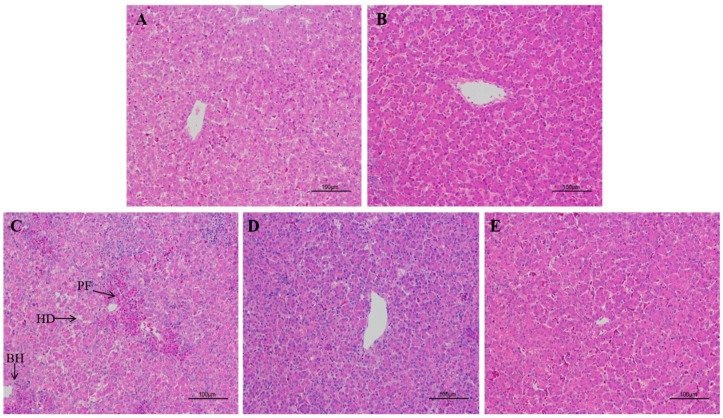
Hepatic histomorphology and histopathology from various groups of experimental broilers. The liver sections were stained with haematoxylin & eosin (100× magnification). (**A**) Control; (**B**) GSPE 250 mg/kg; (**C**) AFB_1_; (**D**) AFB_1_ + GSPE 250 mg/kg; (**E**) AFB_1_ + GSPE 500 mg/kg. PF: periportal fibrosis, HD: hydropic degeneration/fatty changes, and BH: bile duct hyperplasia.

**Figure 3:**
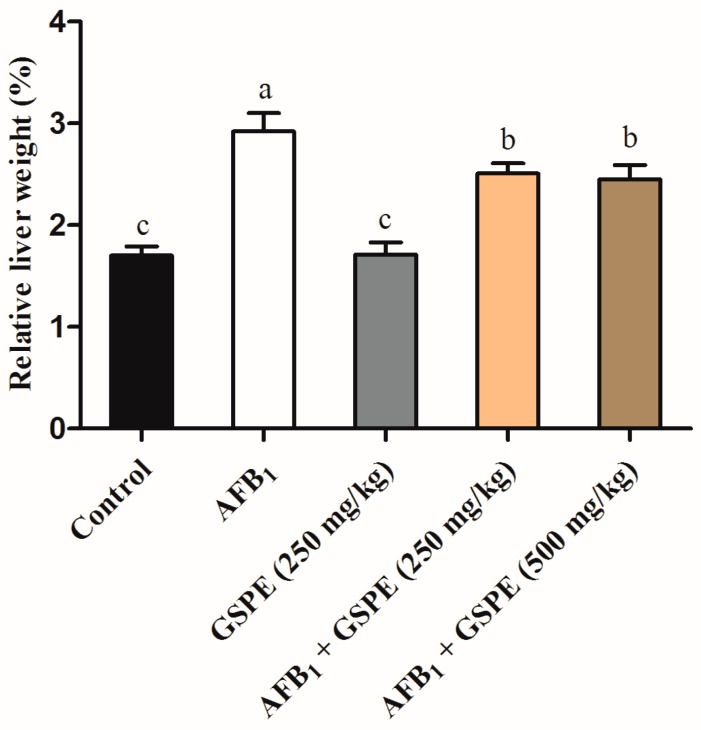
Effects of GSPE on relative liver weight of broilers fed diets contaminated with AFB_1_. Values are represented as the mean ± SD (*n* = 6). ^a–c^ columns with different superscript letters were significantly different (*p* < 0.05). AFB_1_, aflatoxin B_1_; GSPE, grape seed proanthocyanidin extract.

The results of the histopathological changes in the liver are shown in Figure 2. No histopathological alterations were observed in the liver of broilers in the control and GSPE (250 mg/kg) group (Figure 2, profile A, B). In contrast, results of the histological analysis revealed a significant damage in the liver tissue of broilers consumed AFB_1_ alone (Figure 2, profile C). Liver tissue from this treatment had periportal fibrosis, hydropic degeneration/fatty changes, and bile duct hyperplasia when compared with the tissue of birds fed with uncontaminated diet. Strikingly, the supplementation of GSPE (250 or 500 mg/kg) to AFB_1_ diets prevented injury to the hepatic parenchyma of broilers (Figure 2, profiles D, E). Relative liver weight was negatively altered (*p* < 0.05) when birds fed with AFB_1_ contaminated diet (1 mg/kg). The results showed a statistically significant increment in the relative liver weight in the AFB_1_ fed group. The inclusion effect of GSPE (250 or 500 mg/kg) to diet having AFB_1_ (1 mg/kg) can be clearly observed with a significant (*p* < 0.05) improvement in the relative liver weight Figure 3. Moreover, no significant differences were recorded between the two levels of GSPE (250 and 500 mg/kg). Furthermore, histological findings provided additional evidence of the beneficial effect of GSPE on alleviating the toxicity induced by AFB_1_ in broilers.

### 2.6. Hepatic Antioxidant Parameters

The results from the current experiment revealed that feeding broiler with diet contaminated with AFB_1_ (1 mg/kg) has a negative effect (*p* < 0.05) in altering the antioxidant status of the liver Table 4. This effect can be clearly observed with an increment in the content of malondialdehyde (MDA) by 65% as compared with the control group (*p* < 0.05). While the supplementation with GSPE in doses of 250 and 500 mg/kg along with AFB_1_ contaminated diet decreased (*p* < 0.05) MDA content by 45%, 40%, respectively, when compared with the AFB_1_ group.

Moreover, treatments with GSPE (250 and 500 mg/kg) along with AFB_1_ diet resulted in a significant stimulation of the antioxidant system in the liver for counteracting the oxidative damage caused by AFB_1_, resulting in a significant improvement by 31, 29, 23, 24, 17, and 15% in the activity of T-SOD, GSH-Px, CAT, GSH, GR, and GST, respectively, as compared with the AFB_1_ group. In contrast, no significant differences (*p* < 0.05) were recorded for the liver antioxidant parameters excluding GSH-Px and GSH, when GSPE (250 mg/kg) supplemented to uncontaminated diet compared with the control group.

### 2.7. Aflatoxin B_1_ Residues in Liver

**Figure 4:**
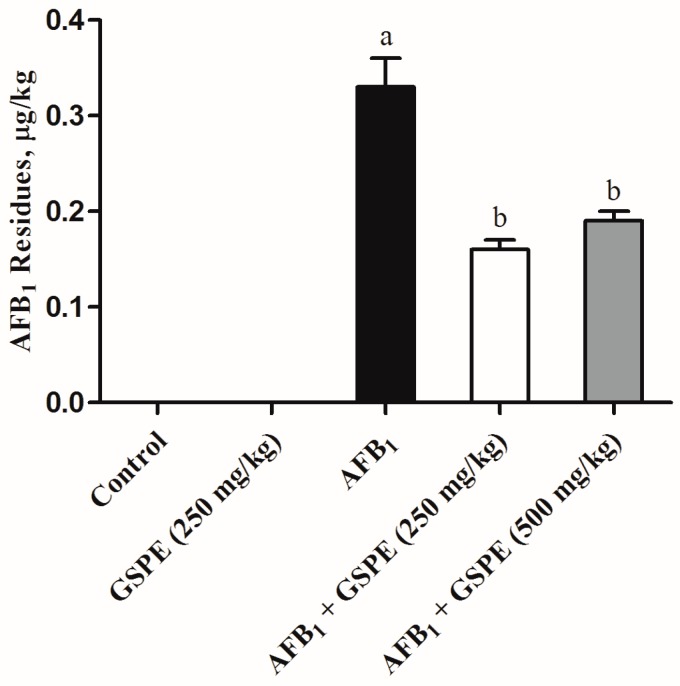
Effects of GSPE on aflatoxin B_1_ residues in liver of broilers fed diet contaminated with AFB_1_. Values are represented as the mean ± SD (*n* = 6). ^a,b^ columns with different superscript letters were significantly different (*p* < 0.05). AFB_1_, aflatoxin B_1_; GSPE, grape seed proanthocyanidin extract.

The AFB_1_ residues in the liver of broilers fed diet contaminated with AFB_1_ or with GSPE are given in Figure 4. AFB_1_ residues were not detectable in the liver of broilers that consumed the uncontaminated diet (control and 250 mg/kg GSPE alone). A detectable level of AFB_1_ (0.33 µg/kg) was found in the liver of broilers fed AFB_1_ contaminated diet. While the supplementation of GSPE (250 and 500 mg/kg) to AFB_1_ diet (1 mg/kg) resulted in a significant decrease in AFB_1_ residues in the liver by 51% and 42%, respectively, when compared with the AFB_1_ alone group.

## 3. Discussion

### 3.1. Growth Performance

AFB_1_ can cause huge economic losses in poultry by reducing growth rate, feed efficiency, and increasing the incidence of disease, hence increasing mortality [10,34]. This study clearly demonstrated the toxic effects of feeding 1 mg/kg AFB_1_ on the growth performance of broiler chickens. These results are in alignment with previous studies, which showed that broilers fed diets contaminated with 1 mg/kg AFB_1_ significantly decreased ADG and ADFI with an adverse effect of AFB_1_ on the cumulative feed gain ratio of broiler chickens when compared with the control group [35,36]. These adverse effects can be explained as AFB_1_ has been associated with reluctance, anorexia, and the inhibition of protein synthesis and lipogenesis [37]. In addition, previous researchers reported that AFB_1_ could alter the intestinal absorbing barrier and reduce the activity of pancreatolipase, amylase, and trypsin, and change the energy metabolism of the cell by disturbing the gluconeogenesis, tricarboxylic acid cycle, and fatty acid synthesis, resulting in lower growth rate [38]. This toxic effect of AFB_1_ on growth performance can be overcome by the supplementation with GSPE. Long, M. et al. [26] reported that GSPE could significantly improve the body weight of mice reduced by AFB_1_. This agrees with our findings, which showed that the addition of GSPE in both levels (250 and 500 mg/kg) to diets contaminated with AFB_1_ significantly improved ADFI, ADG, and FCR when compared with the AFB_1_ group.

### 3.2. Serum Biochemistry

The liver is considered to be the principal target organ for aflatoxins. Determination of the toxic biochemical effects of aflatoxins in serum is necessary for the diagnosis of hepatic damage in broilers [39]. AFB_1_ toxicity in broilers decreases the serum concentration of total protein, albumin, and globulin, and increases hepatic enzyme activities, such as ALT, AST, GGT, and ALP [35,40,41]. Our findings related to serum biochemical changes indicated that feeding diets contaminated with 1 mg/kg AFB_1_ significantly decreased total protein, albumin, and globulin contents, and increased the AST, ALT, GGT, and ALP activities, as compared with the control group. These results are in accordance with previous studies [35,40,41]. The changes in the serum biochemistry during aflatoxicosis could be explained as the protein synthesis in the liver is inhibited, as well as other associated damage in the liver and kidney [41,42]. Furthermore, during the biological conversion of aflatoxins, it produces a large number of active metabolites, which bind to DNA and RNA, resulting in a reduction in the protein production and damaging the liver structure [43,44]. The alteration of AST and ALT may be due to the disruption of the hepatic cells as a result of necrosis or a consequence of altering the cell membrane permeability [45]. Therefore, in our study, the concentration of total protein, albumin, and globulin are significantly increased, and AST, ALT, GGT, and ALP were significantly decreased in serum when broilers ingest aflatoxin B_1_-contaminated diets containing GSPE (250 and 500 mg/kg) as compared with the AFB_1_ group. In this present study, these findings indicated that AFB_1_ damaged the liver and supplementation of GSPE reduced the toxic effects of AFB_1_ on liver functions.

### 3.3. Serum Immunoglobulins 

Toxic effects of AFB_1_ on the immunosuppression in animals is a welfare concern, as it may increase the possibility of the exposure to infectious disease, hence resulting in economic losses [46]. Detecting the concentration of serum immunoglobulins, such as IgA, IgG, and IgM is the most common method to test the humoral immunity response [47]. In the current study, broilers fed diet contaminated with AFB_1_ showed a significant decrease in the content of serum IgA, IgG, and IgM when compared with the control group. These results are in consistent with the previous study that showed that AFB_1_ is known to be immunosuppressive in birds, and reported that a diet containing AFB_1_ significantly reduced the content of serum IgA, IgG, and IgM in broilers [48,49]. These results showed that the humoral function of the body might be impaired by AFB_1_. Nevertheless, studies showed that there was no significant decrease in serum IgM content in broiler chickens that are exposed to AFB_1_ [41,49]. The different results can be explained as the effects of AFB_1_ on humoral immunity depend on the dosage and species of chicken. As AFB_1_ impairs protein synthesis, resulting in a reduction in the contents of immunoglobulins. Furthermore, aflatoxins impair amino acid transport and mRNA transcription, resulting in an inhibition of DNA synthesis, hence reduce antibody titers [43,50]. Besides, the reduction in the frequencies of IgA, IgG, and IgM bearing cells in the bursa of Fabricius induced by aflatoxins, significantly contributed to the reduction of immunoglobulins [51]. Our results showed that the addition of GSPE to contaminated diets with AFB_1_ significantly increased the serum IgA, IgG, and IgM content when compared with the AFB_1_ group. These results indicated that AFB_1_ caused impairment to the immune system. However, the addition of GSPE was able to overcome the adverse effects of AFB_1_ on the immune system.

### 3.4. Serum and Liver Antioxidant Parameters

Recently, there has been increased interest among poultry scientists on the usage of antioxidants against the toxic effects of aflatoxins. This is because aflatoxins have been demonstrated to induce the production of reactive oxygen species (ROS) and oxidative stress can be suggested as one of the underlying mechanisms for AFB_1_ induced cell injury and DNA damage [52]. AFB_1_ increases the production of ROS, consequently attacks the cell membrane lipids, and hence alter the cell membrane fluidity and permeability, resulting in oxidative damage [53,54]. The stage of cell damage and lipid peroxidation can be identified by measuring the content of MDA, which is the main products of polyunsaturated lipid peroxidation [55]. The GSH, SOD, CAT, and GSH-PX are important components of the endogenous antioxidant defense system, play an important role in free radicals scavenging, and maintain the intracellular redox balance. The consumption of AFB_1_ can decrease these antioxidants levels resulting in oxidative stress [56]. Our results showed that diet contaminated with AFB_1_ significantly increased the concentration of MDA and decrease the activities of GSH, T-SOD, CAT, GSH-PX, GR, and GST in the liver and the serum of broilers when compared with the control group. With different doses of AFB_1_, similar toxic effects on oxidative status were observed in the liver and serum of broilers [41,49,57,58]. However, the addition of both levels of GSPE to diets contaminated with AFB_1_ effectively inhibited lipid peroxidation and improved the antioxidant level in the liver and serum. Previous results showed that dietary supplementation of GSPE alleviated AFB_1_-induced oxidative stress and significantly improved the immune injury in mice [26]. Furthermore, the protective action of GSPE against zearalenone induced adverse effects was further demonstrated by improving the antioxidant capacity [25]. Consequently, the antioxidant effect of GSPE in broiler chickens plays a significant role in preventing the oxidative damage that is induced by AFB_1_.

### 3.5. Histopathological Variations and Relative Weight of Liver 

Aflatoxins have several effects on poultry, including liver pathology, and the alterations of relative organ weights [41,49,57]. Histological results revealed that GSPE played a protective role against injuries induced by AFB_1_. Presumably, it has been reported for the first time that GSPE can ameliorate liver injuries in broilers induced by AFB_1_. Intriguingly, dietary supplementation of GSPE mitigated histopathological alterations that were induced by AFB_1_. These findings were similar to the previous study, which showed a positive effect of GSPE to zearalenone-induced hepatic injury [25]. In addition, our results showed that the relative weight of liver significantly increased in the AFB_1_ group when compared with the control group. These results are in consistent with previous studies about the toxicity of AFB_1_ on relative weight of liver, AFB_1_ increases liver weight as lipids accumulate in the liver, which results in hepatomegaly [36,59]. In our present study, the notable increment in liver relative weight in AFB_1_ group was significantly ameliorated by the addition of GSPE (250 and 500 mg/kg) in AFB_1_ contaminated diet. Therefore, these results confirmed that GSPE has a protective effect on the liver damage caused by AFB_1_.

### 3.6. AFB_1_ Residues 

It is well known that the liver is the main detoxifying organ that removes wastes and xenobiotics by metabolic conversion and biliary excretion [60]. It has been demonstrated that aflatoxins intake is associated with a high-level incidence of human liver cancer [61]. In the present study, we observed the level (0.33 µg/kg) of AFB_1_ residues in the liver of broilers fed on the AFB_1_ (1 mg/kg) contaminated diet. Previous research reported that broilers fed AFB_1_ (1 mg/kg) contaminated diet for six weeks, the level of AFB_1_ residues was found (0.166 µg/kg) in the liver [62]. Another study showed that residual level of AFB_1_ (0.05 and 0.13 µg/kg) were also observed in the livers of broilers given diet containing 50 and 100 µg/kg of AFB_1_ [10]. Residues of AFB_1_ were also detected in the liver of laying hens given 2.5 mg/kg of AFB_1_ diet for four weeks, at levels that ranged from 1.92 to 4.13 µg/kg [63]. The differences in the residue levels may be because of the differences in bird and diet types, the concentration of AFB_1_, the duration, and the tolerance to aflatoxins. The results of the present study showed that the level of AFB_1_ residues in the liver significantly decreased with the addition of GSPE to AFB_1_ contaminated diet when compared with the AFB_1_ group. The protective effects of GSPE from AFB_1_ may be due to their specific biotransformation of AFB_1_ in the gastrointestinal tract, which leads to the reduction of AFB_1_ absorption, consequently reduce aflatoxin residues in the liver.

## 4. Conclusions

It can be concluded that dietary supplementation of GSPE (250 and 500 mg/kg) detoxify aflatoxicosis induced by AFB_1_ (1 mg/kg) in broilers, as it improved growth performance, antioxidants capacity, immune function, serum biochemical profile, and histopathological lesions, as well as GSPE resulted in a reduction in the concentration of AFB_1_ residue in the liver. Finally, we suggest that GSPE should be used in either doses 250 or 500 mg/kg as a promising feed additive to detoxify aflatoxins in the broilers feed.

## 5. Material and Methods

### 5.1. Birds, Diets, and Management 

The current study was approved by the Scientific Ethics Committee of Huazhong Agricultural University on 6 March 2016. Ethical approval code HZAUCH-2016-007. Grape seed proanthocyanidin extract was purchased from Zelang Medical Technology Company (Nanjing, China; purity ≥ 95%).

For the experiment, 300 one-day old Cobb broilers were obtained from a commercial hatchery (Jingzhou Kang Poultry Co., Ltd., Jingzhou, China). After three days of acclimation, birds with similar body weight were randomly distributed into five groups with six replicates per group (*n* = 60 per treatment) were grouped based on the following five dietary treatments; 1. A basal diet containing neither GSPE nor AFB_1_ (Control), 2. A basal diet containing 1 mg/kg AFB_1_ from contaminated corn (AFB_1_), 3. Basal diet containing 250 mg/kg GSPE (GSPE 250 mg/kg), 4. Basal diet containing 1 mg/kg AFB_1_ + 250 mg/kg GSPE (AFB_1_ + GSPE 250), 5. Basal diet containing 1 mg/kg AFB_1_ + 500 mg/kg GSPE (AFB_1_ + GSPE 500). Diets and water were provided *ad libitum* during the whole experimental period (4 weeks). The experiment conducted under environmental controlled conditions.

The composition of the basal diets are presented in Table 5.

### 5.2. Aflatoxin B_1_ Production and Analysis

*Aspergillus flavus* strain (NRRL-3357) was used in this study with a known AFB_1_ production capacity [64]. The strain was maintained as a glycerol stock preparation at –80 °C. It was grown on Petri dishes containing potato dextrose agar (E. Merck, Darmstadt, Germany) medium at 30 °C for seven days. Aflatoxin B_1_ was produced according to the technique proposed by Liu Jie et al. [65]. The inoculated maize was incubated for 15 days to obtain the approximate AFB_1_ content of 6400 µg/kg. The AFB_1_ contaminated maize was stored at 4 °C prior to treatment. The aflatoxin B1 concentration was determined by High-Performance Liquid Chromatography (Agilent 1260 series HPLC, Waldbronn, Germany) according to Liu Jie et al. [65].

### 5.3. Collection of Samples and Measurements

Chickens were weighed on a weekly basis and feed consumption for each replicate was measured weekly until the end of the experiment (four weeks). Body weight (BW), average daily feed intake (ADFI), average daily gain (ADG), and feed conversion ratio (FCR) were calculated. At 28 days of age, one bird close to the average weight was selected from each replicate. After the chickens fasted for 12 h, blood samples were collected in tubes by puncture of the wing vein. The blood samples were centrifuged (Eppendorf centrifuge 5804R, Hamburg, Germany) at 1000× *g* at 4 °C for 10 min, and the serum was separated and stored at −20 °C for biochemical, immunoglobulins, and serum antioxidants analysis. After taking blood samples, birds were then sacrificed, the liver was removed and weighed immediately. A portion of liver was snap frozen in liquid nitrogen and stored at −80 °C for antioxidants analysis.

### 5.4. Serum Biochemical and Histopathological Analysis

Serum contents of total protein (TP, g/L), albumin (ALB, g/L), globulin (GLU, g/L), along with activities of aspartate aminotransferase (AST, U/L), alanine aminotransferase (ALT, U/L), gamma-glutamyl transferase (GGT, U/L), and alkaline phosphatase (ALP, U/L) were determined in serum samples. Analysis of the serum samples was performed by an automatic biochemistry analyzer according to the manufacturer’s recommended procedure (Beckman Synchron CX4 PRO, Fullerton, CA, USA). For histopathological examination, the liver tissues were fixed in 10% neutral buffered formalin, routinely embedded in paraffin, cut into 5 µm thick sections, and processed for hematoxylin and eosin (H & E) staining. Liver section from all broilers was microscopically examined.

### 5.5. Serum and Liver Antioxidant Enzymes Assays

Liver tissue samples (0.5 g) were cut into small pieces and homogenized (Ningbo, China) in 4.5 ml ice cold physiological saline. The homogenate was centrifuged at 1000× *g* for 15 min at 4 °C. The supernatant was collected and stored at −80 °C for the following analysis. The activities of catalase (CAT), glutathione peroxide (GSH-Px), total superoxide dismutase (T-SOD), glutathione reductase (GR), and glutathione-S transferase (GST), and content of malondialdehyde (MDA), and glutathione (GSH), in the serum and hepatic supernatants were determined spectrophotometrically (Hengping, Shanghai, China) using commercially available assay kits (Nanjing Jiancheng Bioengineering Institute, Nanjing, China). The details of all the determination procedures followed by the manufacturer’s protocols for the commercial kits.

### 5.6. Serum Concentrations of Immunoglobulins Analysis

The contents of serum, IgA, IgM, and IgG were measured using commercial kits, purchased from (Nanjing Jiancheng Bioengineering Institute, Nanjing, China). The measurements were performed according to the detection kit instructions.

### 5.7. Analysis of Aflatoxin Residues in Liver 

Liver samples were kept at −20 °C for analyzing the residues of AFB_1_. Six birds from each treatment (one bird from each replicate) were selected for this analysis. AFB_1_ residues in liver were analyzed according to the method described by Jie et al. [65] and Ma et al. [66], with slight modification. Briefly, the defrosted liver samples (15 g) were homogenized and blended with 2.5 g of NaCl in 50 mL of methanol and water (80:20) for 30 min. The mixture was filtered through a Whatman filter paper, and then a 10 mL aliquot of the supernatant was collected and mixed with 10 mL Hexane and shaken gently on a mechanical shaker (Shanghai, China) for 15 min. After shaking, the upper layer containing hexane was discarded and the lower layer was separated for further treatment. AFB_1_ was extracted three times with 10, 5, and 5 mL (in total 20 mL) of dichloromethane. The dichloromethane layer was evaporated under the steam of nitrogen at 60 °C.

The residue obtained after the evaporation was re-dissolved in 200 µL of acetonitrile/water (9:1, *v*/*v*) and was then derivatized using 700 µL of TFA (trifluoroacetic acid)/acetic acid/water (20:10:70, *v*/*v*/*v*). The derivatized solution was filtered through Millex PTFE 0.22 µm filters (Tianjin, China). Finally, samples were analyzed using an Agilent 1260 series HPLC (Waldbronn, Germany) equipped with a C_18_ column (250 × 4.6 mm, 5 µm, Agilent). A mobile phase composed of water: methanol: acetonitrile (60:30:10), was used in this study at a flow rate of 1 mL/min. Detection was performed by fluorescence detector at a wavelength, excitation 360 nm, and emission 440 nm.

### 5.8. Statistical Analysis

One-way ANOVA was applied to assess the differences among mean values. Duncan’s test was used for multiple comparisons when a significant difference was detected. All data are presented as the mean ± SD, and the significance level was set at *p* < 0.05 for all measurements. Whole analysis was conducted using IBM SPSS Statistic 22 (IBM Corporation, Armonk, New York, NY, USA).

## Figures and Tables

**Table 1:** Effects of grape seed proanthocyanidin extract (GSPE) on growth performance of broilers fed diets contaminated with Aflatoxin B_1_ (AFB_1_).

Parameters	Dietary Treatments				
	Control	AFB_1_ (1 mg/kg)	GSPE (250 mg/kg)	AFB_1_ + GSPE (250 mg/kg)	AFB_1_ + GSPE (500 mg/kg)
1–2 weeks					
ADFI (g/day)	52.56 ± 0.97 ^a^	40.31 ± 1.54 ^c^	51.7 ± 1.19 ^a^	45.35 ± 1.21 ^b^	45.91 ± 2.70 ^b^
ADG (g/day)	39.09 ± 1.43 ^a^	25.77 ± 1.31 ^c^	38.92 ± 1.44 ^a^	30.85 ± 0.82 ^b^	30.61 ± 1.99 ^b^
FCR (feed:gain)	1.35 ± 0.05 ^c^	1.57 ± 0.13 ^a^	1.33 ± 0.04 ^c^	1.47 ± 0.04 ^b^	1.50 ± 0.05 ^a,b^
3–4 weeks					
ADFI (g/day)	104.95 ± 9.16 ^a^	71.64 ± 4.42 ^c^	106.24 ± 2.44 ^a^	80.72 ± 2.62 ^b^	83.74 ± 1.62 ^b^
ADG (g/day)	69.69 ± 2.90 ^b^	45.14 ± 3.98 ^d^	73.78 ± 1.27 ^a^	53.13 ± 1.57 ^c^	54.08 ± 1.74 ^c^
FCR (feed:gain)	1.51 ± 0.12 ^a,b^	1.59 ± 0.07 ^a^	1.44 ± 0.02 ^b^	1.52 ± 0.08 ^a,b^	1.55 ± 0.03 ^a^
1–4 weeks					
ADFI (g/day)	78.75 ± 4.78 ^a^	55.98 ± 2.79 ^c^	78.80 ± 1.02 ^a^	63.04 ± 1.17 ^b^	64.82 ± 2.07 ^b^
ADG (g/day)	54.40 ± 1.75 ^b^	35.46 ± 1.71 ^d^	56.43 ± 1.25 ^a^	41.99 ± 1.10 ^c^	42.35 ± 1.66 ^c^
FCR (feed:gain)	1.45 ± 0.07 ^c^	1.58 ± 0.03 ^a^	1.40 ± 0.02 ^c^	1.50 ± 0.06 ^b^	1.53 ± 0.02 ^a,b^

Values are represented as the mean ± SD (*n* = 60). ^a–d^ Mean values within a row with different superscript letters were significantly different (*p* < 0.05). AFB_1_, aflatoxin B_1_; GSPE, grape seed proanthocyanidin extract; ADFI, average daily feed intake; ADG, average daily gain; FCR, feed conversion ratio.

**Table 2:** Effects of GSPE on serum biochemical parameters of broilers fed diets contaminated with AFB_1_.

Parameters	Dietary Treatments				
	Control	AFB_1_	GSPE	AFB_1_ + GSPE	AFB_1_ + GSPE
		(1 mg/kg)	(250 mg/kg)	(250 mg/kg)	(500 mg/kg)
ALT (U/L)	1.76 ± 0.13 ^b^	2.43 ± 0.18 ^a^	1.68 ± 0.10 ^b^	1.87 ± 0.14 ^b^	1.85 ± 0.17 ^b^
AST (U/L)	272.57 ± 24.34 ^c^	352.63 ± 30.23 ^a^	257.53 ± 14.37 ^c^	312.25 ± 19.67 ^b^	298.67 ± 16.50 ^b^
GGT (U/L)	20.45 ± 1.37 ^c^	29.57 ± 1.69 ^a^	20.95 ± 1.43 ^c^	26.38 ± 1.85 ^b^	24.62 ± 1.65 ^b^
ALP (U/L)	1499.75 ± 73.22 ^c^	1891.43 ± 144.70 ^a^	1481.93 ± 88.92 ^c^	1730.88 ± 136.48 ^b^	1608.38 ± 124.54 ^b,c^
TP (g/L)	29.57 ± 2.07 ^a^	18.32 ± 1.43 ^c^	29.45 ± 1.01 ^a^	24.57 ± 2.10 ^b^	23.08 ± 1.86 ^b^
Albumin (g/L)	15.50 ± 0.96 ^a^	10.33 ± 0.65 ^c^	15.92 ± 0.88 ^a^	13.05 ± 0.94 ^b^	13.13 ± 0.82 ^b^
Globulin (g/L)	12.57 ± 0.43 ^a^	9.01 ± 0.65 ^c^	12.61 ± 0.63 ^a^	11.82 ± 0.61 ^b^	12.02 ± 0.52 ^a,b^

Values are represented as the mean ± SD (*n* = 6). ^a–c^ Mean values within a row with different superscript letters were significantly different (*p* < 0.05). AFB_1_, aflatoxin B_1_; GSPE, grape seed proanthocyanidin extract; ALT, alanine aminotransferase; AST, aspartate aminotransferase; GGT, gamma-glutamyl transferase; ALP, alkaline phosphate; TP, total protein.

**Table 3:** Effects of GSPE on antioxidant parameters in the serum of broilers fed diets contaminated with AFB_1_.

Parameters	Dietary Treatments				
	Control	AFB_1_	GSPE	AFB_1_ + GSPE	AFB_1_ + GSPE
		(1 mg/kg)	(250 mg/kg)	(250 mg/kg)	(500 mg/kg)
MDA, nmol/mL	2.46 ± 0.19 ^c^	4.05 ± 0.44 ^a^	2.32 ± 0.18 ^c^	3.24 ± 0.32 ^b^	3.37 ± 0.29 ^b^
T-SOD, U/mL	157.52 ± 13.20 ^b^	108.91 ± 10.55 ^c^	182.57 ± 9.47 ^a^	150.53 ± 11.02 ^b^	154.29 ± 9.35 ^b^
GSH-Px, U/mL	1544.56 ± 62.50 ^a^	913.67 ± 82.97 ^c^	1629.54 ± 133.29 ^a^	1293.92 ± 100.44 ^b^	1345.37 ± 88.06 ^b^
CAT, U/mL	2.80 ± 0.24 ^a,b^	1.83 ± 0.17 ^d^	2.91 ± 0.25 ^a^	2.59 ± 0.16 ^b,c^	2.36 ± 0.21 ^c^
GSH, mg/L	5.76 ± 0.23 ^b^	3.02 ± 0.18 ^c^	7.26 ± 0.63 ^a^	6.24 ± 0.47 ^b^	5.93 ± 0.40 ^b^
GR, U/L	27.39 ± 2.75 ^b^	16.67 ± 1.43 ^d^	31.28 ± 2.64 ^a^	24.17 ± 1.55 ^c^	25.08 ± 2.26 ^b,^^c^
GST, U/mL	57.25 ± 3.00 ^a^	39.76 ± 3.50 ^c^	54.81 ± 4.48 ^a,b^	50.31 ± 4.03 ^b^	51.95 ± 3.69 ^b^

Values are represented as the mean ± SD (*n* = 6). ^a–d^ Mean values within a row with different superscript letters were significantly different (*p* < 0.05). AFB_1_, aflatoxin B_1_; GSPE, grape seed proanthocyanidin extract; MDA, malondialdehyde; T-SOD, total superoxide dismutase; GSH-Px, glutathione peroxidase; CAT, catalase; GSH, glutathione; GR, glutathione reductase; GST, glutathione S-transferase.

**Table 4:** Effects of GSPE on antioxidant parameters in the liver of broilers fed diets contaminated with AFB_1_.

Parameters		Dietary Treatments			
	Control	AFB_1_	GSPE	AFB_1_ + GSPE	AFB_1_ + GSPE
		(1 mg/kg)	(250 mg/kg)	(250 mg/kg)	(500 mg/kg)
MDA, nmol/mgprot	0.96 ± 0.05 ^c^^,d^	1.59 ± 0.17 ^a^	0.82 ± 0.10 ^d^	1.09 ± 0.08 ^b,^^c^	1.14 ± 0.13 ^b^
T-SOD, U/mgprot	71.87 ± 4.59 ^a^	52.70 ± 7.04 ^b^	79.19 ± 2.75 ^a^	76.14 ± 6.67 ^a^	76.52 ± 8.50 ^a^
GSH-Px, U/mgprot	51.65 ± 4.66 ^b^	29.77 ± 3.56 ^d^	62.46 ± 5.94 ^a^	40.29 ± 3.54 ^c^	43.81 ± 4.98 ^c^
CAT, U/mgprot	86.79 ± 9.14 ^a^	57.47 ± 9.12 ^c^	95.46 ± 8.85 ^a^	75.22 ± 5.16 ^b^	73.34 ± 6.42 ^b^
GSH, mg/gprot	2.83 ± 0.18 ^b^	2.09 ± 0.20 ^c^	3.10 ± 0.22 ^a^	2.78 ± 0.18 ^b^	2.71 ± 0.23 ^b^
GR, U/gprot	6.65 ± 0.65 ^a^	3.18 ± 0.30 ^c^	7.03 ± 0.48 ^a^	4.04 ± 0.42 ^b^	3.68 ± 0.34 ^b,^^c^
GST, U/mgprot	25.28 ± 1.44 ^a^	19.03 ± 1.48 ^c^	26.49 ± 2.25 ^a^	21.96 ± 1.46 ^b^	22.82 ± 1.77 ^b^

Values are represented as the mean ± SD (*n* = 6). ^a–d^ Mean values within a row with different superscript letters were significantly different (*p* < 0.05). AFB_1_, aflatoxin B_1_; GSPE, grape seed proanthocyanidin extract; MDA, malondialdehyde; T-SOD, total superoxide dismutase; GSH-Px, glutathione peroxidase; CAT, catalase; GSH, glutathione; GR, glutathione reductase; GST, glutathione S-transferase.

**Table 5:** Basal diet formulations and nutritional contents.

**Ingredients**	**Percentage %**
Corn	58.3
Soybean meal	30.2
Fish meal	5.6
Soybean oil	2.3
Dicalcium phosphate	1.2
Lime stone	1.00
Salt	0.2
Methionine	0.2
Premix ^1^	1.00
Total	100.00
**Calculated chemical composition**	
Crude protein	21.87
Metabolisable energy (MJ/kg)	13.45
Lysine	1.14
Methionine	0.40
Methionine + Cystine	0.94
Calcium	0.95
Available phosphorus	0.49

^1^ The premix contained (per kg of diet): Fe, 60 mg; Cu, 7.5 mg; Zn, 65 mg; Mn, 110 mg; I, 1.1 mg; Se, 0.4 mg; Biotin, 0.04 mg; choline chloride, 400 mg; vitamin A (from retinyl acetate), 4500 IU; vitamin D3 (from cholecalciferol), 1000 IU; vitamin K (menadione sodium bisulphate), 1.3 mg; vitamin B1, 2.2 mg; vitamin B2, 10 mg; vitamin B3, 10 mg; vitamin B5, 50 mg; vitamin B6, 4 mg; vitamin B11, 1 mg; vitamin B12, 0.013 mg.

## References

[1] Diaz D.E., Hagler W.M., Hopkins B.A., Whitlow L.W. (2003). Aflatoxin binders I: In vitro binding assay for aflatoxin B1 by several potential sequestering agents. Mycopathologia.

[2] Alpsoy L., Yalvac M.E. (2010). Key roles of vitamins A, C, and E in aflatoxin B1-induced oxidative stress. Vitam. Horm..

[3] Dalvi R. (1986). An overview of aflatoxicosis of poultry: Its characteristics, prevention and reduction. Vet. Res. Commun..

[4] Kermanshahi H., Hazegh A., Afzali N. (2009). Effect of sodium bentonite in broiler chickens fed diets contaminated with aflatoxin B_1_. J. Anim. Vet. Adv..

[5] Magnoli A., Monge M., Miazzo R., Cavaglieri L., Magnoli C., Merkis C., Cristofolini A., Dalcero A., Chiacchiera S. (2011). Effect of low levels of aflatoxin B_1_ on performance, biochemical parameters, and aflatoxin B_1_ in broiler liver tissues in the presence of monensin and sodium bentonite. Poult. Sci..

[6] Surai P.F. (2002). Natural Antioxidants in Avian Nutrition and Reproduction.

[7] Shen H.-M., Shi C.-Y., Lee H.-P., Ong C.-N. (1994). Aflatoxin B1-induced lipid peroxidation in rat liver. Toxicol. Appl. Pharmacol..

[8] Aflatoxins (2012). IARC.

[9] Hussain Z., Khan M.Z., Khan A., Javed I., Saleemi M.K., Mahmood S., Asi M.R. (2010). Residues of aflatoxin B1 in broiler meat: Effect of age and dietary aflatoxin B1 levels. Food Chem. Toxicol..

[10] Bintvihok A., Kositcharoenkul S. (2006). Effect of dietary calcium propionate on performance, hepatic enzyme activities and aflatoxin residues in broilers fed a diet containing low levels of aflatoxin B1. Toxicon.

[11] Zhang N.-Y., Qi M., Zhao L., Zhu M.-K., Guo J., Liu J., Gu C.-Q., Rajput S.A., Krumm C.S., Qi D.-S. (2016). Curcumin prevents aflatoxin B1 hepatoxicity by inhibition of cytochrome p450 isozymes in chick liver. Toxins (Basel).

[12] Li Y., Ma Q., Zhao L., Guo Y., Duan G., Zhang J., Ji C. (2014). Protective efficacy of alpha-lipoic acid against aflatoxinB1-induced oxidative damage in the liver. Asian-Australas. J. Anim. Sci..

[13] Abdel-Hamid A.A., Firgany A.E.-D.L. (2015). Vitamin e supplementation ameliorates aflatoxin B1-induced nephrotoxicity in rats. Acta Histochem..

[14] Zhen J., Qu Z., Fang H., Fu L., Wu Y., Wang H., Zang H., Wang W. (2014). Effects of grape seed proanthocyanidin extract on pentylenetetrazole-induced kindling and associated cognitive impairment in rats. Int. J. Mol. Med..

[15] El-Ashmawy I.M., Saleh A., Salama O.M. (2007). Effects of marjoram volatile oil and grape seed extract on ethanol toxicity in male rats. Basic Clin. Pharmacol. Toxicol..

[16] Sharma S.D., Katiyar S.K. (2010). Dietary grape seed proanthocyanidins inhibit uvb-induced cyclooxygenase-2 expression and other inflammatory mediators in uvb-exposed skin and skin tumors of SKH-1 hairless mice. Pharm. Res..

[17] Bagchi D., Sen C.K., Ray S.D., Das D.K., Bagchi M., Preuss H.G., Vinson J.A. (2003). Molecular mechanisms of cardioprotection by a novel grape seed proanthocyanidin extract. Mutat. Res..

[18] Demirkaya E., Avci A., Kesik V., Karslioglu Y., Oztas E., Kismet E., Gokcay E., Durak I., Koseoglu V. (2009). Cardioprotective roles of aged garlic extract, grape seed proanthocyanidin, and hazelnut on doxorubicin-induced cardiotoxicity. Can. J. Physiol. Pharmacol..

[19] Ahn S.-H., Kim H.J., Jeong I., Hong Y.J., Kim M.-J., Rhie D.-J., Jo Y.-H., Hahn S.J., Yoon S.H. (2011). Grape seed proanthocyanidin extract inhibits glutamate-induced cell death through inhibition of calcium signals and nitric oxide formation in cultured rat hippocampal neurons. BMC Neurosci..

[20] Liu P., Kemper L.J., Wang J., Zahs K.R., Ashe K.H., Pasinetti G.M. (2011). Grape seed polyphenolic extract specifically decreases aβ* 56 in the brains of tg2576 mice. J. Alzheimers Dis..

[21] Sayin V.I., Ibrahim M.X., Larsson E., Nilsson J.A., Lindahl P., Bergo M.O. (2014). Antioxidants accelerate lung cancer progression in mice. Sci. Transl. Med..

[22] Ouédraogo M., Charles C., Ouédraogo M., Guissou I.P., Stévigny C., Duez P. (2011). An overview of cancer chemopreventive potential and safety of proanthocyanidins. Nutr. Cancer.

[23] Bagchi D., Bagchi M., Stohs S.J., Das D.K., Ray S.D., Kuszynski C.A., Joshi S.S., Pruess H.G. (2000). Free radicals and grape seed proanthocyanidin extract: Importance in human health and disease prevention. Toxicology.

[24] Xu Z.-C., Yin J., Zhou B., Liu Y.-T., Yu Y., Li G.-Q. (2015). Grape seed proanthocyanidin protects liver against ischemia/reperfusion injury by attenuating endoplasmic reticulum stress. World J. Gastroenterol..

[25] Long M., Yang S.-H., Han J.-X., Li P., Zhang Y., Dong S., Chen X., Guo J., Wang J., He J.-B. (2016). The protective effect of grape-seed proanthocyanidin extract on oxidative damage induced by zearalenone in kunming mice liver. Int. J. Mol. Sci..

[26] Long M., Zhang Y., Li P., Yang S.-H., Zhang W.-K., Han J.-X., Wang Y., He J.-B. (2016). Intervention of grape seed proanthocyanidin extract on the subchronic immune injury in mice induced by aflatoxin B1. Int. J. Mol. Sci..

[27] Dai N., Zou Y., Zhu L., Wang H.-F., Dai M.-G. (2014). Antioxidant properties of proanthocyanidins attenuate carbon tetrachloride (CCL4)–induced steatosis and liver injury in rats via CYP2E1 regulation. J. Med. Food.

[28] Sano T., Oda E., Yamashita T., Naemura A., Ijiri Y., Yamakoshi J., Yamamoto J. (2005). Anti-thrombotic effect of proanthocyanidin, a purified ingredient of grape seed. Thromb. Res..

[29] Engelbrecht A.-M., Mattheyse M., Ellis B., Loos B., Thomas M., Smith R., Peters S., Smith C., Myburgh K. (2007). Proanthocyanidin from grape seeds inactivates the PI3-kinase/PKB pathway and induces apoptosis in a colon cancer cell line. Cancer Lett..

[30] Liu X., Qiu J., Zhao S., You B., Ji X., Wang Y., Cui X., Wang Q., Gao H. (2012). Grape seed proanthocyanidin extract alleviates ouabain-induced vascular remodeling through regulation of endothelial function. Mol. Med. Rep..

[31] Gao Z., Liu G., Hu Z., Li X., Yang X., Jiang B., Li X. (2014). Grape seed proanthocyanidin extract protects from cisplatin-induced nephrotoxicity by inhibiting endoplasmic reticulum stress-induced apoptosis. Mol. Med. Rep..

[32] Al-Sowayan N.S., Kishore U. (2012). Prophylactic efficacy of a combination of proanthocyanidin and vitamin e on hepatotoxicity induced by doxorubicin in rats. Int. Res. J. Pharm..

[33] Mansouri E., Khorsandi L., Moaiedi M.Z. (2015). Grape seed proanthocyanidin extract improved some of biochemical parameters and antioxidant disturbances of red blood cells in diabetic rats. Iran. J. Pharm. Res..

[34] Ortatatli M., Oğuz H., Hatipoğlu F., Karaman M. (2005). Evaluation of pathological changes in broilers during chronic aflatoxin (50 and 100 ppb) and clinoptilolite exposure. Res. Vet. Sci..

[35] Gowda N.K., Ledoux D.R., Rottinghaus G.E., Bermudez A.J., Chen Y.C. (2009). Antioxidant efficacy of curcuminoids from turmeric (Curcuma longa L.) powder in broiler chickens fed diets containing aflatoxin B1. Br. J. Nutr..

[36] Gowda N., Ledoux D., Rottinghaus G., Bermudez A., Chen Y. (2008). Efficacy of turmeric (curcuma longa), containing a known level of curcumin, and a hydrated sodium calcium aluminosilicate to ameliorate the adverse effects of aflatoxin in broiler chicks. Poult. Sci..

[37] Bagherzadeh Kasmani F., Karimi Torshizi M., Allameh A., Shariatmadari F. (2012). A novel aflatoxin-binding bacillus probiotic: Performance, serum biochemistry, and immunological parameters in japanese quail. Poult. Sci..

[38] Osborne D., Huff W., Hamilton P., Burmeister H. (1982). Comparison of ochratoxin, aflatoxin, and t-2 toxin for their effects on selected parameters related to digestion and evidence for specific metabolism of carotenoids in chickens 1, 2. Poult. Sci..

[39] Abdel-Wahhab M., Nada S., Amra H. (1999). Effect of aluminosilicates and bentonite on aflatoxin-induced developmental toxicity in rat. J. Appl. Toxicol..

[40] Oguz H., Kececi T., Birdane Y., Önder F., Kurtoglu V. (2000). Effect of clinoptilolite on serum biochemical and haematological characters of broiler chickens during aflatoxicosis. Res. Vet. Sci..

[41] Shi Y., Xu Z., Feng J., Wang C. (2006). Efficacy of modified montmorillonite nanocomposite to reduce the toxicity of aflatoxin in broiler chicks. Anim. Feed Sci. Technol..

[42] Wu J.-W., Lin L.-C., Hung S.-C., Lin C.-H., Chi C.-W., Tsai T.-H. (2007). Hepatobiliary excretion of silibinin in normal and liver cirrhotic rats. Drug Metab. Dispos..

[43] Yunus A.W., Razzazi-Fazeli E., Bohm J. (2011). Aflatoxin B1 in affecting broiler’s performance, immunity, and gastrointestinal tract: A review of history and contemporary issues. Toxins (Basel).

[44] Miazzo R., Peralta M., Magnoli C., Salvano M., Ferrero S., Chiacchiera S., Carvalho E., Rosa C., Dalcero A. (2005). Efficacy of sodium bentonite as a detoxifier of broiler feed contaminated with aflatoxin and fumonisin. Poult. Sci..

[45] Ozer J., Ratner M., Shaw M., Bailey W., Schomaker S. (2008). The current state of serum biomarkers of hepatotoxicity. Toxicology.

[46] Fink-Gremmels J. (2008). The role of mycotoxins in the health and performance of dairy cows. Vet. J..

[47] Meissonnier G.M., Pinton P., Laffitte J., Cossalter A.-M., Gong Y.Y., Wild C.P., Bertin G., Galtier P., Oswald I.P. (2008). Immunotoxicity of aflatoxin B1: Impairment of the cell-mediated response to vaccine antigen and modulation of cytokine expression. Toxicol. Appl. Pharmacol..

[48] Chen K., Fang J., Peng X., Cui H., Chen J., Wang F., Chen Z., Zuo Z., Deng J., Lai W. (2014). Effect of selenium supplementation on aflatoxin B_1_-induced histopathological lesions and apoptosis in bursa of fabricius in broilers. Food Chem. Toxicol..

[49] Liu T., Ma Q., Zhao L., Jia R., Zhang J., Ji C., Wang X. (2016). Protective effects of sporoderm-broken spores of ganderma lucidum on growth performance, antioxidant capacity and immune function of broiler chickens exposed to low level of aflatoxin B1. Toxins (Basel).

[50] Thaxton J., Tung H., Hamilton P. (1974). Immunosuppression in chickens by aflatoxin 1, 2. Poult. Sci..

[51] Ul-Hassan Z., Zargham Khan M., Khan A., Javed I. (2012). Immunological status of the progeny of breeder hens kept on ochratoxin a (OTA)-and aflatoxin B1 (AFB_1_)-contaminated feeds. J. Immunotoxicol..

[52] Yang C.-F., Liu J., Wasser S., Shen H.-M., Tan C.E.-L., Ong C.-N. (2000). Inhibition of ebselen on aflatoxin B1-induced hepatocarcinogenesis in fischer 344 rats. Carcinogenesis.

[53] Kodama M., Inoue F., Akao M. (1990). Enzymatic and non-enzymatic formation of free radicals from aflatoxin B1. Free Radic. Res. Commun..

[54] Choi K.-C., Lee B.-S., Chung W.-T., Choi M.-S., Lee J.-C. (2010). Protective effects of apigenin and quercetin on aflatoxin B_1_-induced immunotoxicity in mice. Food Sci. Biotechnol..

[55] Naaz F., Abdin M., Javed S. (2014). Protective effect of esculin against prooxidant aflatoxin B1-induced nephrotoxicity in mice. Mycotoxin Res..

[56] Yener Z., Celik I., Ilhan F., Bal R. (2009). Effects of urtica dioica l. Seed on lipid peroxidation, antioxidants and liver pathology in aflatoxin-induced tissue injury in rats. Food Chem. Toxicol..

[57] Fan Y., Zhao L., Ji C., Li X., Jia R., Xi L., Zhang J., Ma Q. (2015). Protective effects of bacillus subtilis ansb060 on serum biochemistry, histopathological changes and antioxidant enzyme activities of broilers fed moldy peanut meal naturally contaminated with aflatoxins. Toxins (Basel).

[58] Zuo R.-Y., Chang J., Yin Q.-Q., Wang P., Yang Y.-R., Wang X., Wang G.-Q., Zheng Q.-H. (2013). Effect of the combined probiotics with aflatoxin B_1_-degrading enzyme on aflatoxin detoxification, broiler production performance and hepatic enzyme gene expression. Food Chem. Toxicol..

[59] Chen X., Horn N., Applegate T. (2014). Efficiency of hydrated sodium calcium aluminosilicate to ameliorate the adverse effects of graded levels of aflatoxin B1 in broiler chicks. Poult. Sci..

[60] Taub R. (2004). Liver regeneration: From myth to mechanism. Nat. Rev. Mol. Cell Biol..

[61] Berry C.L. (1988). The pathology of mycotoxins. J. Pathol..

[62] Denli M., Blandon J., Guynot M., Salado S., Perez J. (2009). Effects of dietary afladetox on performance, serum biochemistry, histopathological changes, and aflatoxin residues in broilers exposed to aflatoxin B1. Poult. Sci..

[63] Zaghini A., Martelli G., Roncada P., Simioli M., Rizzi L. (2005). Mannanoligosaccharides and aflatoxin B1 in feed for laying hens: Effects on egg quality, aflatoxins B1 and M1 residues in eggs, and aflatoxin B1 levels in liver. Poult. Sci..

[64] Schmidt-Heydt M., Abdel-Hadi A., Magan N., Geisen R. (2009). Complex regulation of the aflatoxin biosynthesis gene cluster of aspergillus flavus in relation to various combinations of water activity and temperature. Int. J. Food Microbiol..

[65] Liu J., Sun L., Zhang N., Zhang J., Guo J., Li C., Rajput S.A., Qi D. (2016). Effects of nutrients in substrates of different grains on aflatoxin B1 production by aspergillus flavus. Biomed. Res. Int..

[66] Ma H., Zhang N., Sun L., Qi D. (2015). Effects of different substrates and oils on aflatoxin B1 production by aspergillus parasiticus. Eur. Food Res. Technol..

