# Correction: Rajput, S.A., et al. Ameliorative Effects of Grape Seed Proanthocyanidin Extract on Growth Performance, Immune Function, Antioxidant Capacity, Biochemical Constituents, Liver Histopathology and Aflatoxin Residues in Broilers Exposed to Aflatoxin B_1_. *Toxins* 2017, *9*, 371

**DOI:** 10.3390/toxins10090366

**Published:** 2018-09-10

**Authors:** Shahid Ali Rajput, Lvhui Sun, Niya Zhang, Mahmoud Mohamed Khalil, Xin Gao, Zhao Ling, Luoyi Zhu, Farhan Anwar Khan, Jiacai Zhang, Desheng Qi

**Affiliations:** 1 Department of Animal Nutrition and Feed Science, College of Animal Science and Technology, Huazhong Agricultural University, Wuhan 430070, China; dr.shahidali@hotmail.com (S.A.R.); lvhuisun@mail.hzau.edu.cn (L.S.); zhangniya@mail.hzau.edu.cn (N.Z.); mahmoud.khalil@fagr.bu.edu.eg (M.M.K.); gaoxinnutrition@webmail.hzau.edu.cn (X.G.); lingzhao@webmail.hzau.edu.cn (Z.L.); zhuluoyi@outlook.com (L.Z.); hznyzjc@hotmail.com (J.Z.); 2 Animal Production Department, Faculty of Agriculture, Benha University, Moshtohor, Benha, Kalubia 13736, Egypt; 3 Department of Animal Health, Faculty of Animal Husbandry and Veterinary Sciences, University of Agriculture, Peshawar 25120, Pakistan; farhan82@aup.edu.pk

The authors wish to make the following correction to their paper [1].

There is one typing mistake in Section 5.2. The current version is “The inoculated maize was incubated for 15 days to obtain the approximate AFB_1_ content of 6400 µg/kg”. The correct version should be “The inoculated maize was incubated for 15 days to obtain the approximate AFB_1_ content of 64 mg/kg”.

The changes do not affect the scientific results. The manuscript will be updated and the original will remain online on the article webpage. We apologize for any inconvenience caused to our readers.
